# Food insecurity as a predictor of all-cause mortality and premature mortality among older adults: a longitudinal cohort analysis of ELSA study

**DOI:** 10.1007/s40520-026-03422-3

**Published:** 2026-05-27

**Authors:** Vincenza Gianfredi, Daniele Nucci, Pinar Soysal, Stefania Maggi, Alberto Castagna, Andrea Cozza, Vincenzo Baldo, Nicola Veronese

**Affiliations:** 1 Department of Cardiac, Thoracic, Vascular Sciences, and Public Health, University of Padua, via Loredan, 18, 35122 Padua, Italy; 2 Struttura Semplice Dipartimentale Igiene Alimenti e Nutrizione, Dipartimento di Igiene e Prevenzione Sa-nitaria, Agenzia di Tutela della Salute (ATS) Brescia, Via Duca degli Abruzzi, 15, Brescia, 25124 Italy; 3 PhD National Program in One Health Approaches to Infectious Diseases and Life Science Research De-partment of Public Health, Experimental and Forensic Medicine, University of Pavia, Pavia, 27100 Italy; 4 Department of Geriatric Medicine, Faculty of Medicine, Bezmialem Vakif University, Istanbul, Turkey; 5 National Research Council (CNR), Aging Section, Padova, Italy; 6 Department of Primary Care, Health District of Soverato, Azienda Sanitaria Provinciale Catanzaro, Soverato, Italy; 7 Faculty of Medicine, Saint Camillus International University of Health Sciences, Rome, Italy

**Keywords:** Food insecurity, Mortality, Premature mortality, Older adults

## Abstract

**Background:**

Food insecurity is a growing public health concern, particularly among older adults, as it has been linked to adverse health outcomes, including chronic diseases, sarcopenia and functional decline. However, its direct association with all-cause and premature mortality remains underexplored.

**Aims:**

This study aims to evaluate the relationship between food insecurity and mortality among individuals aged 50 years and older using data from the English Longitudinal Study of Ageing (ELSA).

**Methods:**

A longitudinal analysis was conducted using data from ELSA wave 2 (2004–2005), including 8,686 participants aged ≥ 50 years with available information on food insecurity. Food insecurity was assessed through a single-item self-reported measure. All-cause and early mortality were determined using linked mortality records. Cox proportional hazards models were used to estimate hazard ratios (HRs) and 95% confidence intervals (CIs), adjusting for key sociodemographic, lifestyle, and health-related confounders.

**Results:**

During the follow-up period, food insecurity was significantly associated with a 41% higher risk of all-cause mortality (HR = 1.41, 95% CI: 1.04–2.82, *p* = 0.035) after full adjustment for confounders. Additionally, food insecurity was linked to a 126% increased risk of premature mortality (HR = 2.26, 95% CI: 1.26–4.02, *p* = 0.006).

**Discussion:**

Findings suggest that addressing food insecurity could represent a novel strategy to prevent sarcopenia and related mortality in older adults.

**Conclusions:**

Given its significant public health implications, targeted interventions are essential to reduce food insecurity and its associated health burden, ultimately improving longevity and quality of life among aging populations.

**Supplementary Information:**

The online version contains supplementary material available at 10.1007/s40520-026-03422-3.

## Introduction

Food insecurity—the inability to access sufficient, safe, and nutritious food to meet dietary needs for an active and healthy life—remains a pressing public health issue with significant implications for morbidity and mortality worldwide [1]. It is a multidimensional construct influenced by socioeconomic, environmental, and policy factors, disproportionately affecting vulnerable populations, including older adults [2]. However, in large epidemiological studies, food insecurity is often assessed using simplified indicators that may capture only specific dimensions of this multidimensional construct. Older adults are particularly vulnerable to food insecurity due to a combination of physiological, economic, and social factors [3]. Age-related changes, such as reduced appetite, altered taste perception, and difficulties with chewing or swallowing, can compound the effects of inadequate food access [4]. Additionally, many older adults live on fixed incomes, making them more susceptible to financial strain and reducing their ability to purchase sufficient nutritious food [5]. Limited mobility, lack of transportation, and physical disabilities further restrict access to food retailers, particularly for those living in food deserts or in rural areas [6]. Social isolation, which is prevalent among older individuals [7], exacerbates food insecurity by limiting access to support networks that might otherwise help with food acquisition and meal preparation [8]. The global burden of food insecurity has increased in recent years due to economic instability, rising living costs, and health crises, exacerbating health disparities and contributing to adverse health outcomes [9, 10].

A growing body of evidence suggests that food insecurity is a major determinant of poor health. It has been linked to an increased risk of chronic diseases such as cardiovascular disease [11], diabetes [12], and mental health disorders [13], as well as to functional decline and frailty in older adults [14]. Among the physiological consequences of poor dietary intake and nutritional inadequacy, sarcopenia plays a pivotal role in mediating the relationship between food insecurity and mortality. Sarcopenia—defined by the progressive decline in skeletal muscle mass, strength, and performance—is exacerbated by inadequate protein and micronutrient intake, systemic inflammation, and reduced physical activity, all common in food-insecure older adults [15, 16]. Recent studies have identified sarcopenia as a key determinant of disability, hospitalization, and death in aging populations, suggesting it may act as an intermediary pathway linking food insecurity with premature mortality [17, 18]. Epidemiological studies have demonstrated that food-insecure older adults exhibit significantly lower intakes of essential nutrients, a dietary pattern associated with heightened susceptibility to chronic diseases such as cardiovascular disease, osteoporosis, and diabetes [19, 20]. Beyond direct nutritional deficiencies, the psychosocial stress associated with food insecurity can contribute to systemic inflammation, impaired immune function, and dysregulation of metabolic pathways, further heightening the risk of mortality [21].

Despite the well-documented associations between food insecurity and health outcomes [22], evidence on its relationship with mortality remains limited and not fully consistent. Some longitudinal studies conducted in general adult populations, particularly in North America, have reported an association between food insecurity and increased risk of all-cause mortality [23, 24]. However, these studies often focus on broader age ranges and do not specifically address older adults, who may be particularly vulnerable due to the interplay of nutritional, physiological, and social factors.

Furthermore, the relationship between food insecurity and premature mortality has received comparatively little attention, especially in European populations. To date, few studies have examined this association using longitudinal data in aging cohorts, limiting the understanding of how food insecurity may influence early mortality risk in later life. In addition, differences in healthcare systems, social protection policies, and population characteristics may affect the generalizability of findings across settings. The English Longitudinal Study of Ageing (ELSA), a nationally representative cohort of older adults in England, provides a unique opportunity to investigate this association. Given that food insecurity is often intertwined with broader social determinants of health—including economic instability, social isolation, and access to healthcare—understanding its role in mortality risk can inform targeted public health interventions and policies aimed at mitigating its consequences. To our knowledge, this is among the few studies to examine the association between food insecurity and both all-cause and premature mortality in a large, nationally representative cohort of older adults in Europe, providing novel insights into the long-term health consequences of food insecurity in this population.

The present study aims to address these gaps by assessing the association between food insecurity and all-cause mortality, as well as premature mortality, in a large cohort of older adults, while accounting for key sociodemographic, lifestyle, and health-related factors. By evaluating this relationship while accounting for key confounders such as sociodemographic factors, lifestyle behaviors, and multimorbidity, this study seeks to contribute to the growing evidence base on food insecurity as an important driverof health outcomes and longevity.

## Materials and methods

### Study population

**Fig. 1:**
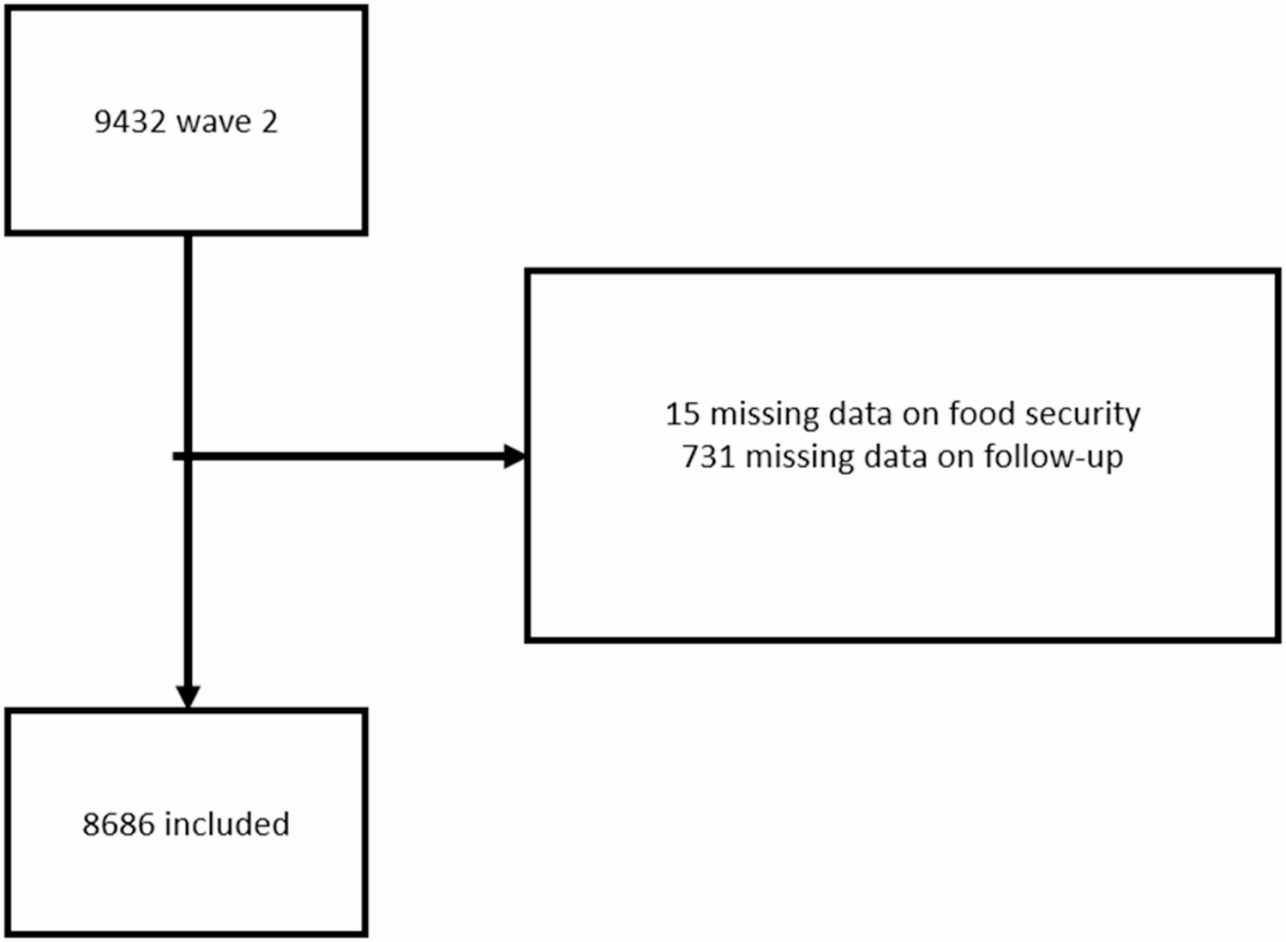
Flow chart

The English Longitudinal Study of Ageing (ELSA) is an ongoing, nationally representative cohort study that follows adults residing in England. This study utilized data from the second wave of ELSA (2004–2005), which included 9,432 participants. For the purposes of this analysis, only individuals aged over 50 years were considered. Complete baseline data on food insecurity (with 15 missing cases, 0.2%) and lost at follow-up (with 731 missing, 7.7%) were available for 8,686 participants, representing 92.1% of the original wave 2 cohort (Fig. 1). The 731 with missing data during follow-up about mortality were similarly in mean age (*p* = 0.23) and prevalence of females (*p* = 0.65). The ELSA study received ethical approval from the London Multicenter Research Ethics Committee (MREC/01/2/91), and all participants provided written informed consent.

### Exposure: food insecurity

In the ELSA study, food insecurity was assessed through a single-item question asking participants whether they had ever been forced to reduce or skip meals due to financial constraints with the following question: “Have meals ever had to be cut or skipped as there was not enough money for food?”. Response options included “yes,” “no,” “not applicable,” and “unknown”. The changes in food insecurity during the follow-up period was used as time dependent covariate in the analyses. This measure primarily captures the quantitative and economic dimension of food insecurity—specifically, reduced food intake due to financial constraints—and does not encompass other dimensions such as food quality, nutritional adequacy, food safety, or stability over time.

### Outcomes: mortality and premature mortality

Premature mortality was defined as death occurring before a predefined age threshold, in line with established public health conventions. In this study, we operationalized premature mortality as death occurring before the age of 75 years, consistent with commonly used benchmarks in epidemiological and health policy research. This cutoff reflects widely adopted definitions in high-income countries and is supported by national statistics from the United Kingdom, where it is used as an indicator of avoidable or early mortality. Given that life expectancy in England exceeds 80 years, the threshold of 75 years was selected to capture deaths occurring substantially earlier than the expected lifespan, thereby identifying premature mortality in a standardized and policy-relevant manner [25]. In this context, premature mortality reflects deaths occurring before age 75 during the available follow-up period, rather than the cumulative lifetime probability of dying before that age. Given the age distribution of the cohort and the 10-year follow-up duration, not all participants had the same opportunity to reach the age threshold of 75 years during observation.

### Confounders

Covariates were selected based on their previously established associations with both the exposure and outcomes [26]. The variables included were: age (measured in years, treated as a continuous variable); sex; race (Whites vs. others); years of education (also considered as a continuous variable); smoking history (categorized as ever vs. never smoker); and marital status (classified as married vs. all other statuses, including partnered, separated, divorced, widowed, or never married). Physical activity levels were determined based on self-reported engagement in moderate exercise over the past week and dichotomized into sedentary (including no, low and moderate physical activity), or high activity level. This variable was derived from the combination of the frequency of vigorous (s2vgactx_e), moderate (s2mdactx_e), and light (s2ltactx_e) physical activity during the week. Information on chronic medical conditions was obtained through self-reported physician diagnoses, covering high blood pressure, diabetes, cancer, lung disease, heart disease, stroke, psychiatric disorders, arthritis, asthma, high cholesterol, cataracts, Parkinson’s disease, hip fractures, Alzheimer’s disease, and other dementias. The total number of chronic conditions was summed, and multimorbidity was defined as the presence of two or more chronic conditions, following established criteria [27, 28]. Depressive symptoms were assessed using an eight-item version of the CES-D [29]. Study participants were asked about the occurrence (yes/no) of eight depressive symptoms such as feeling depressed or feeling sad for much of the time over the previous week to give a possible maximum score of 8. Obesity was defined using a body mass index (BMI) value over 30 Kg/m^2^ [30], with weight and height recorded by a trained nurse. Finally, disability was evaluated as having at least one difficulty in activities of daily living (ADL) versus no difficulty. Covariates were selected based on their established associations with both the exposure and the outcome. These included sociodemographic, lifestyle, and health-related variables. Some variables, such as multimorbidity, obesity, and physical inactivity, may represent baseline health status but could also lie on the causal pathway between food insecurity and mortality. Therefore, their inclusion in fully adjusted models should be interpreted cautiously, as it may lead to partial over-adjustment.

### Statistical analyses

Means and standard deviations (SD) were used to describe quantitative measures, while percentages and counts were used for categorical variables. Characteristics of the study participants at baseline (wave 2) were compared according to the presence or not of food insecurity, using the Chi-square/Fisher exact tests for categorical variables, and a T-test for independent groups, after testing for homoscedasticity of the variances with the Levene test, for continuous variables.

The association between food insecurity at baseline and all-cause mortality during the follow-up was explored by survival curves using Kaplan-Meier analyses: since it was not violated, as graphically reported in Supplementary Fig. 1, a Cox’s regression analysis was applied. Cox proportional hazard models were used to estimate hazard ratios (HR) and 95% confidence intervals (95% CI) for the association between food insecurity at baseline and all-cause mortality. We included all the covariates significantly different across food insecurity status at baseline (*p* < 0.05) or associated with all-cause mortality during follow-up (*p* < 0.10). Given the potential for certain covariates to act as mediators rather than pure confounders, the fully adjusted models should be interpreted as estimating associations conditional on these factors, rather than total causal effects. The collinearity among covariates was assessed using the variance inflation factor, taking a value over two as exclusion criterion. However, no parameter was excluded for this reason. We repeated the same elaborations taking premature mortality as outcome.

All statistical tests were two-tailed, and a p-value < 0.05 was considered to be statistically significant. All analyses were performed using SPPS 26.0.

## Results

### Study population characteristics

**Fig. 2:**
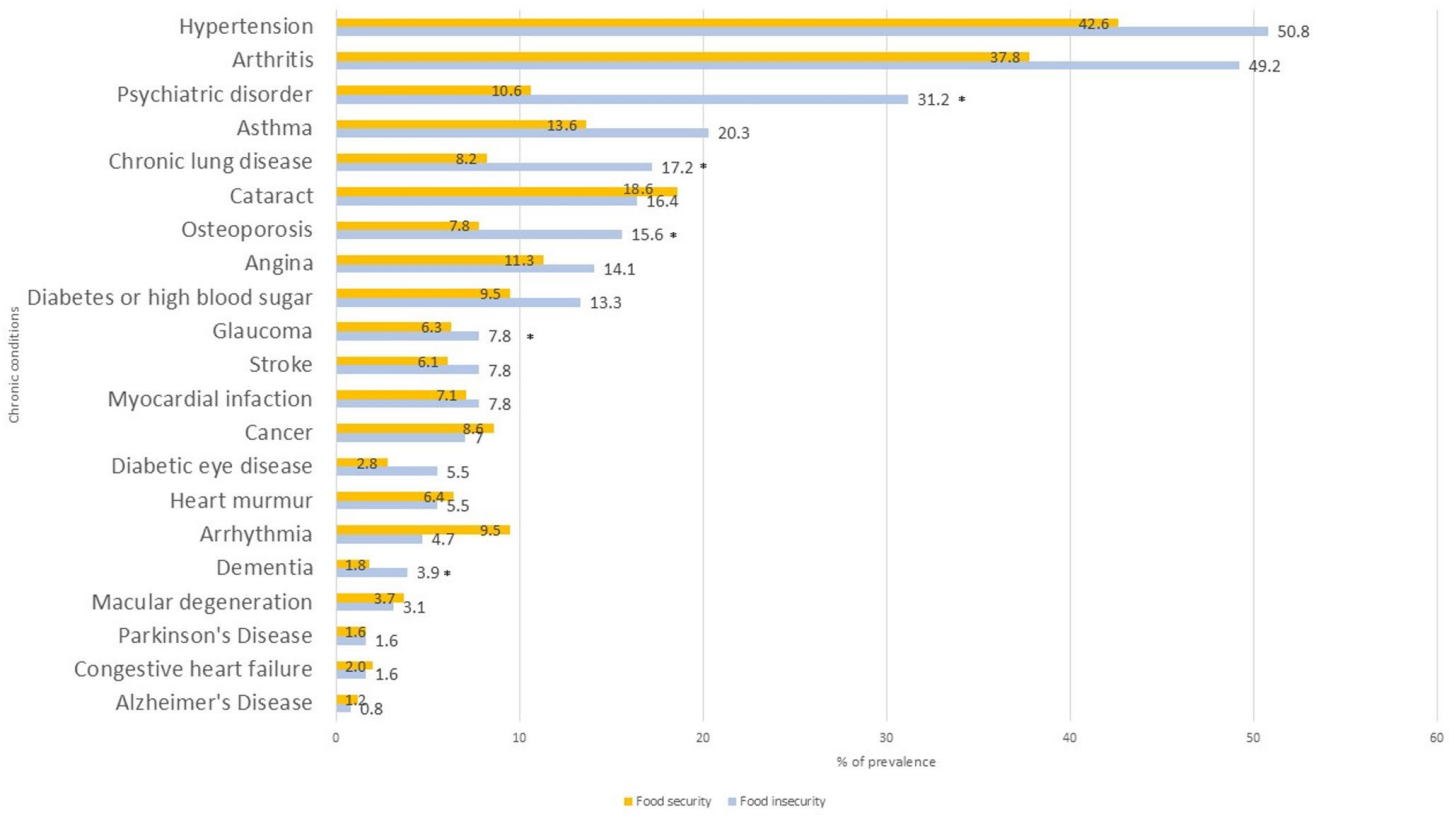
Distribution of chronic diseases between food insecurity and mortality participants. * statistically significant. Chi-square test

A total of 8,686 participants from the ELSA cohort were included in this analysis, of whom 128 (1.5%) reported experiencing food insecurity, while the remaining 8,558 participants were classified as food secure. Participants experiencing food insecurity were significantly younger (*p* < 0.001), had lower levels of education (*p* = 0.002), were less likely to be married (*p* < 0.001), and had a higher prevalence of multimorbidity (*p* < 0.001) compared to food-secure individuals (Table 1). Food-insecure participants were also more sedentary (*p* < 0.001), had lower alcohol consumption (*p* < 0.001), and a higher prevalence of obesity (*p* = 0.010). Additionally, they reported a significantly higher burden of depressive symptoms as measured by CESD (*p* < 0.001) and greater difficulty with activities of daily living (*p* < 0.001). Participants experiencing food insecurity exhibited a significantly higher prevalence of chronic lung disease (*p* = 0.001), osteoporosis (*p* = 0.006), psychiatric disorders (*p* < 0.001), dementia (*p* < 0.001), and glaucoma (*p* = 0.042) compared to their food-secure counterparts. Results are shown in Fig. 2, and supplementary Table 1.

**Table 1:** Descriptive characteristics of the participants (*n* = 8,686), stratified by food security level. Food security has been assessed with a single question: “Have meals ever had to be cut or skipped as there was not enough money for food” (yes/no)

Parameter	Food insecurity (*n* = 128)	Food security (*n* = 8558)	*p*-value
Age (mean, SD)	61.3 (8.77)	65.9 (10.47)	< 0.001
Female gender (%)	61.7%	56.2%	0.209
Race (white) (%)	89.8%	97.9%	< 0.001
Years of education (mean, SD)	5.0 (6.59)	7.7 (7.00)	0.002
Married (%)	28.1%	66.8%	< 0.001
Sedentary behaviour (%)	47.7%	31.6%	< 0.001
Ever smoked (%)	20.3%	37.4%	< 0.001
Multimorbidity (%)	66.4%	46.0%	< 0.001
Alcohol intake (%)	32.8%	57.6%	< 0.001
Obesity (%)	31.3%	22.3%	0.010
Diff ADLs	1.0 (1.33)	0.4 (0.91)	< 0.001
CESD	3.9 (2.68)	1.6 (1.92)	< 0.001
Food insecurity (%)			
Severe Moderate Mild Low	40.6% 21.9% 21.1% 16.4%	Not applicable	Not applicable

Severe: one or more times per month; moderate: almost every month; mild: most months but not every month; low: once or twice in the year

During a follow-up of 10 years, a total of 2142 all-cause deaths and 735 premature deaths were documented for a total of 62,559 person-years. The Supplementary Fig. 1 presents Kaplan-Meier survival curves comparing the survival probability over a 10-year follow-up period between individuals experiencing food insecurity and those who are food secure. The survival probability declines more rapidly in the food-insecure group, indicating a higher mortality risk compared to their food-secure counterparts. At 5 years of follow-up, the estimated survival probability was approximately 95.7% among food-secure individuals compared to about 90.5% among those experiencing food insecurity. At 10 years, survival probabilities were approximately 93.0% and 84.5%, respectively, corresponding to an absolute difference of about 8.5% points between groups. This suggests that food insecurity is associated with lower survival rates among older adults.

In the unadjusted Cox regression model, food insecurity was associated with a 95% increased risk of all-cause mortality (HR = 1.95, 95% CI: 1.20–3.19, *p* = 0.008). After adjusting for potential confounders—including age, gender, education, marital status, smoking, sedentary behavior, alcohol intake, obesity, multimorbidity, functional limitations, depressive symptoms, and race—this association remained statistically significant, with food insecurity increasing the hazard of mortality by 41% (HR = 1.41, 95% CI: 1.04–2.82, *p* = 0.035).

When assessing premature mortality, no significant association was observed in the unadjusted model (HR = 1.30, 95% CI: 0.67–2.50, *p* = 0.440). However, after full adjustment, food insecurity was associated with a 126% higher hazard of premature mortality (HR = 2.57, 95% CI: 1.32–5.03, *p* = 0.006), suggesting that food-insecure individuals may experience disproportionately higher rates of premature death (Table 2).

**Table 2:** Association between food insecurity and mortality

Outcome	Incidence rate (per 1,000 persons-year) (95% CI)	Crude model (HR, 95% CI)	*p*-value	Fully-adjusted model^1^ (HR, 95% CI)	*p*-value
**Mortality**	34 (32–37)	1.95 (1.20–3.19)	0.008	1.41 (1.04–2.82)	0.035
**Premature mortality**	12 (11–13)	1.30 (0.67–2.50)	0.440	2.57 (1.32–5.03)	0.006

^1^Fully-adjusted model included: age (as continuous variable); gender; years of education (as continuous variable); marital status (married vs. other status); smoking status (ever vs. never); sedentary behavior (low physical activity and sedentary vs. moderate and vigorous physical activity); alcohol; obese, presence of multimorbidity (yes/no), activity of daily living (as continuous variable), CESD (as continuous variable), race (white vs. non-white)

## Discussion

The relatively low prevalence of food insecurity observed in this cohort (1.4%) should be interpreted in light of the study population and measurement approach. In particular, the use of a single-item measure focusing on severe manifestations of food insecurity (i.e., skipping or reducing meals due to financial constraints) may underestimate its true prevalence. Nevertheless, the findings of this study provide evidence that food insecurity is significantly associated with increased all-cause mortality and premature mortality among older adults. Even after adjusting for key sociodemographic, lifestyle, and health-related confounders, food insecurity remained a strong predictor of mortality, emphasizing its role as an independent risk factor for adverse health outcomes in aging populations. The use of a standardized threshold for premature mortality (i.e., 75 years) enhances comparability with public health indicators, although alternative cutoffs may be considered depending on the population context.

The observed association between food insecurity and mortality aligns with previous research indicating that food-insecure individuals are at greater risk of chronic disease, functional decline, and poor mental health, all of which contribute to increased mortality risk [22–24]. Notably, food-insecure participants in our study exhibited a higher burden of multimorbidity, greater functional limitations, and a higher prevalence of depressive symptoms. This underscores the complex interplay between nutritional, physiological, and psychosocial determinants of health, which must be considered when designing public health strategies to address food insecurity. The observed association between food insecurity and increased mortality risk is consistent with previous evidence. A recent systematic review and meta-analysis of large-scale cohort studies reported that food insecurity is associated with a significantly higher risk of all-cause mortality [31]. The magnitude of association observed in our study (HR = 1.41 for all-cause mortality) is broadly in line with these pooled estimates, supporting the robustness of this relationship across different populations and study designs.

Food insecurity also promotes chronic inflammation and hormonal dysregulation, which are recognized contributors to sarcopenia progression [32]. A key methodological consideration relates to the role of certain covariates included in the fully adjusted models. Variables such as multimorbidity, obesity, and physical inactivity may act not only as confounders but also as mediators in the pathway linking food insecurity to mortality. Adjusting for such variables may introduce over-adjustment bias or collider stratification bias, potentially attenuating the observed associations. Therefore, the estimates from fully adjusted models should be interpreted with caution, as they may represent conservative estimates of the total effect. Future studies adopting a causal inference framework, including the use of directed acyclic graphs (DAGs) and mediation analyses, are warranted to better disentangle these complex relationships. Importantly, the measure of food insecurity used in this study reflects reduced food intake due to financial constraints and does not directly capture dietary quality, nutritional adequacy, or food safety. Therefore, interpretations related to nutritional mechanisms should be considered indirect and inferred rather than directly observed.

The mechanisms linking food insecurity to mortality can be broadly distinguished into those supported by empirical evidence and those that remain more speculative or indirectly inferred. Food insecurity in older adults is a multidimensional risk factor for mortality, operating through complex biological pathways involving nutritional deficiencies, disease exacerbation, systemic inflammation, metabolic dysfunction, and psychosocial stress. Among the most well-supported mechanisms, inadequate intake has been consistently associated with frailty [33], muscle wasting [14], osteoporosis [34], and a weakened immune system [35], all of which contribute to increased vulnerability to adverse health outcomes and mortality. Similarly, limited access to nutrient-dense foods is well-documented to exacerbate chronic diseases by limiting the intake of high-quality proteins, essential amino acids, and micronutrients crucial for muscle maintenance and repair [36, 37]. Other pathways, while biologically plausible, remain less directly established. For instance, food-insecure may contribute to chronic low grade inflammation through poor diet quality and psychosocial stress, potentially involving levels of pro-inflammatory cytokines such as C-reactive protein (CRP), interleukin-6 (IL-6), and tumor necrosis factor-alpha (TNF-α) [21, 38]. Additionally, activation of the hypothalamic-pituitary-adrenal (HPA) axis and sustained cortisol elevation could partially explain links with metabolic and neurodegenerative diseases such as dementia [39].Cyclical patterns of food deprivation and compensatory overeating [40]have also been hypothesized to influence weight fluctuations and metabolic dysregulation [41], central adiposity [42], which in turn may increase burden of metabolic disorders [26]. These conditions are major contributors to cardiovascular mortality, the leading cause of death among older adults [43]. However, these pathways require further longitudinal and mechanistic investigation to establish causality.

The findings of this study have important implications for public health policy, particularly within the United Kingdom, where food insecurity among older adults is an increasingly recognized concern. Evidence suggests that food insecurity in the UK is closely linked to socioeconomic disadvantage, social isolation, and barriers in accessing support services, particularly among older populations [44]. Within the context of the UK’s National Health Service and social care system, addressing food insecurity requires coordinated action across healthcare, social services, and community-based organizations. Given that food insecurity is a modifiable risk factor, targeted interventions and policy initiatives can play a crucial role in reducing its impact on mortality and premature mortality in aging populations [45]. Addressing food insecurity requires a multifaceted and multisectoral approach, integrating efforts from healthcare systems, social services, and public health programs to ensure older adults have access to adequate, nutritious food and supportive resources [46].

From a policy perspective, expanding and strengthening food assistance programs—such as meal delivery services, food subsidy programs, and community-based nutrition initiatives—can improve food security and dietary quality among vulnerable older adults [47]. Additionally, efforts to integrate food security screening into routine healthcare practice, particularly in primary care and geriatric settings, could facilitate early identification and intervention for at-risk individuals. Healthcare professionals should be trained to recognize food insecurity as a relevant driver of health outcomes and to connect affected individuals with appropriate resources. There is growing evidence supporting the integration of food insecurity screening into routine clinical practice, particularly in primary care settings, as a strategy to improve patient outcomes and facilitate access to social and nutritional support services [22]. Nutritional interventions ensuring adequate protein intake, combined with physical activity promotion, should be integrated into food assistance and aging programs [48].

Future research should focus on evaluating the longitudinal effects of food insecurity interventions, identifying the most effective strategies for mitigating its impact on health and mortality. Additionally, exploring the role of nutritional supplementation, behavioral interventions, and psychosocial support in reducing the health risks associated with food insecurity could inform future public health strategies. Expanding research efforts to diverse populations and settings will also be essential to tailor interventions to specific socioeconomic and cultural contexts.

### Limitations and strengths

This study has several limitations that should be considered when interpreting the findings. First, food insecurity was assessed using a single self-reported question, which may not fully capture the complexity and multidimensional nature of the construct. Food insecurity encompasses several dimensions, including not only the quantitative insufficiency of food, but also aspects related to food quality, psychological stress, and social acceptability. The use of a single-item measure may therefore lead to misclassification and potentially underestimate the true prevalence and severity of food insecurity. Moreover, this approach limits comparability with studies employing validated multi-item instruments. Second, although we adjusted for a wide range of potential confounders, the possibility of residual confounding cannot be entirely excluded. In particular, unmeasured or incompletely captured factors—such as dietary quality, social support networks, access to healthcare services, and food environment characteristics—may influence both food insecurity and mortality risk. Third, the study population was drawn from the ELSA, a cohort of older adults residing in England, which may limit the generalizability of the findings to other populations with different socioeconomic and healthcare contexts. Differences in socioeconomic conditions, healthcare systems, social protection policies, and food assistance programs across countries may influence both the prevalence of food insecurity and its relationship with health outcomes. Therefore, the observed associations may not be directly transferable to settings with different structural and cultural contexts. Additionally, the reliance on self-reported chronic conditions and lifestyle factors introduces the potential for measurement bias. Lastly, while this study establishes an association between food insecurity and mortality, causal inferences should be made with caution, as observational studies cannot definitively establish causality.

Despite these limitations, this study has several notable strengths. It leverages data from a large, nationally representative cohort, providing robust evidence on the long-term health consequences of food insecurity in older adults. The use of longitudinal data with a long follow-up period strengthens the validity of the findings by allowing for the assessment of temporal relationships between food insecurity and mortality. Additionally, comprehensive statistical adjustments for sociodemographic, lifestyle, and health-related variables enhance the reliability of the results. Lastly, the study also contributes to the limited body of literature exploring the impact of food insecurity on premature mortality, highlighting a critical and underexplored public health issue.

## Conclusions

In conclusion, this study adds to the growing body of evidence demonstrating the far-reaching impact of food insecurity on health and survival in later life. Given the increasing prevalence of food insecurity among aging populations, particularly in the context of economic instability and rising living costs, urgent action is needed to address this pressing public health challenge. Our findings suggest that food insecurity may contribute to mortality in older adults through mechanisms involving malnutrition. From a policy and clinical perspective, integrating routine screening for food insecurity into healthcare practice—particularly in primary care and geriatric settings—could facilitate early identification of at-risk individuals and enable timely referral to social and nutritional support services. Tackling food insecurity could therefore serve as a strategy to preserve muscle health, reduce frailty, and improve longevity. Future research should focus on identifying effective intervention strategies, understanding the role of modifiable risk factors, and exploring policy-based solutions to reduce food insecurity and its associated mortality burden in older adults.

## Supplementary Information

Below is the link to the electronic supplementary material.

Supplementary Material 1

## Data Availability

The original data presented in the study are openly available in ELSA PROJECT, available at https://www.elsa-project.ac.uk/.
