# Supplementary material for: Food insecurity as a predictor of all-cause mortality and premature mortality among older adults: a longitudinal cohort analysis of ELSA study
Source: Aging Clin Exp Res. 2026 May 27;38(1):166. doi: 10.1007/s40520-026-03422-3 (PMC13415261; doi:10.1007/s40520-026-03422-3)

Supplementary Table 1. **Distribution of chronic diseases between food insecurity and food security participants.**

| **Chronic diseases** | **Food insecurity** | **Food security** | **p-value§** |
| --- | --- | --- | --- |
| Hypertension (%) | 50.8 | 42.6 | 0.368 |
| Angina (%) | 14.1 | 11.3 | 0.724 |
| Myocardial infaction (%) | 7.8 | 7.1 | 0.940 |
| Congestive heart failure (%) | 1.6 | 2.0 | 0.305 |
| Heart murmur (%) | 5.5 | 6.4 | 0.971 |
| Arrhythmia (%) | 4.7 | 9.5 | 0.213 |
| Diabetes or high blood sugar (%) | 13.3 | 9.5 | 0.479 |
| Stroke (%) | 7.8 | 6.1 | 0.398 |
| Chronic lung disease (%) | 17.2 | 8.2 | 0.001* |
| Asthma (%) | 20.3 | 13.6 | 0.142 |
| Arthritis (%) | 49.2 | 37.8 | 0.057 |
| Osteoporosis (%) | 15.6 | 7.8 | 0.006* |
| Cancer (%) | 7.0 | 8.6 | 0.798 |
| Parkinson's Disease (%) | 1.6 | 1.6 | 0.878 |
| Psychiatric disorder (%) | 31.2 | 10.6 | <0.001* |
| Alzheimer's Disease (%) | 0.8 | 1.2 | 0.963 |
| Dementia (%) | 3.9 | 1.8 | <0.001* |
| Glaucoma (%) | 7.8 | 6.3 | 0.042* |
| Diabetic eye disease (%) | 5.5 | 2.8 | 0.061 |
| Macular degeneration (%) | 3.1 | 3.7 | 0.290 |
| Cataract (%) | 16.4 | 18.6 | 0.891 |

* statistically significant; § chi-square test

**Supplementary figure 1. Kaplan-Meier Survival Curves Stratified by Food Insecurity (red line, "No"; blue line, "Yes"), over a 10-year follow-up.**


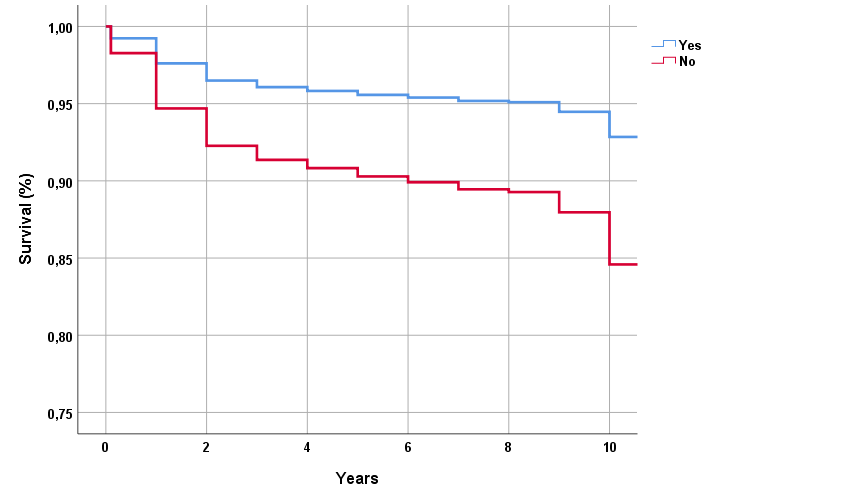

Supplement: Supplementary file 1 — Supplementary Material 1 [file 40520_2026_3422_MOESM1_ESM.docx]
